# Prehospital time intervals for trauma patients according to population density levels in Sweden; a national retrospective cohort study

**DOI:** 10.1186/s13049-025-01514-z

**Published:** 2025-12-04

**Authors:** Oscar H. M. Lundberg, Oscar Lapidus, Denise Bäckström

**Affiliations:** 1 Department of Intensive and Perioperative Care, Skåne University Hospital, Malmö, Sweden; 2 Department of Clinical Sciences (IKVL), Anaesthesiology and Intensive Care, Medical Faculty, Lund University, Lund, Sweden; 3 Department of Clinical Science, Intervention and Technology (CLINTEC), Karolinska Institutet, Stockholm, Sweden; 4 Department of Biomedical and Clinical Sciences (BKV), Faculty of Medicine and Health Sciences, Linköping University, Linköping, Sweden; 5 Department of Leadership and Command & Control, Swedish Defence University, Karlstad, Sweden

**Keywords:** Trauma, Transportation of patients, Emergency medical services, Response time, On-scene time, Transport time, Population density, Prehospital care, Mortality

## Abstract

**Background:**

Minimising time from injury to hospital admission is considered a key factor in trauma. Trauma care is often centralised to hospitals, which, because of their urban location, make treatment more accessible to patients in densely populated areas. If prehospital time increases with declining population density, an effect on mortality could hence be present. The primary aim of this study was to describe prehospital time intervals across population density groups. A secondary objective was to compare the 30-day mortality rates in these population groups.

**Methods:**

This retrospective cohort study was based on the Swedish Trauma registry (SweTrau) between 2018 and 2019. Based on their home municipality, patients were divided into groups of high, medium or low population density. The time interval distributions were described and compared. Secondary outcomes were reported. A multivariate mortality analysis included time intervals, demographics, injury severity score, physiological parameters and other covariables such as care provided by a prehospital physician.

**Results:**

A total of 14,538 patients were included. The distribution across high, medium and low population concentrations was 34%, 47% and 19%, respectively. The response and transport times were significantly longer in the low population group compared with patients from groups high and medium, with a median difference of 4 and 11–15 min, respectively (*p* < 0.001). The median on-scene time of 20 min was shortest in the medium group with a one minute difference to both other groups (*p* < 0.001). The crude mortality of 5% in the low density group was significantly lower than in the other two (both 6%) (*p* = 0.005). However, after adjustment no association between mortality and prolonged prehospital time intervals was seen. The involvement of a prehospital physician in the care was associated with lower mortality (OR 0.60, 95% CI 0.39–0.91; *p* = 0.02).

**Conclusions:**

Although prehospital time intervals increased with remoteness, these differences were not associated with increased mortality. Further, residing in either high, medium or low density population areas was not consistently associated with mortality.

**Supplementary Information:**

The online version contains supplementary material available at 10.1186/s13049-025-01514-z.

## Background

Violence and injuries are accountable for nearly half a million deaths in Europe per year, and are the leading causes of death among people aged 15–29 years [1]. Most of these deaths occur at the scene or before hospitalization [2, 3]. Shortening of transport times does hence seem plausible to prevent trauma related deaths. The widespread concept of “the golden hour”, first attributed to Cowley, where critically ill patients within 60 min should be offered definitive care, has deeply influenced prehospital care, but does, however, not seem to rely on explicit research findings [4].

Both prolonged on-scene-time and response time have been shown to be associated with dying according to some authors [5–7], while others have not been able to show the same [8–12]. In subgroups of patients with hemodynamic instability due to penetrating trauma or with traumatic brain injury, swift transport to hospital seems beneficial [13]. On the other hand, interventions as blood transfusions given prehospitally [14], sedation and intubation of comatose patients before admission to hospital also seem beneficial [15], raising the question if what really matters for some patients are specific treatments and not necessary where these are given.

Population density, geographical differences, the corresponding access to advanced medical treatment and the overall effects of these factors on prehospital time intervals and mortality among trauma patients have been sparsely investigated. A recent article by Nilsbakken et al. [16] reported longer prehospital time intervals in rural areas compared to urban areas in a Norwegian setting, but remoteness was itself not associated with mortality. O´Reilly and colleagues found population density not being associated with mortality among trauma patients in Ireland, but did not report prehospital times [17]. The, to our knowledge, latest description of trauma patients in the Swedish prehospital system regarding population density and survival, did also not report prehospital time intervals [18].

The primary aim of this retrospective cohort study was to compare prehospital time intervals for trauma patients, including a separate analysis of the more severe cases, across different population density levels in Sweden.

Secondary objectives included to investigate whether the population density level was associated with mortality, length of stay (LOS) and Glasgow outcome scale (GOS). Further, we wanted to investigate what factors were associated with mortality after adjustments in a multivariate model.

We hypothesized prehospital time intervals would be longer in areas with lower population density and mortality would increase with longer transport times.

## Methods

### Study design

This study employed a retrospective observational design to conduct a comprehensive comparison of prehospital times for a cohort of injured trauma patients based on their home addresses in municipalities with high, medium or low population density. The primary objective was to explore potential disparities in transport times based on population density. Ethical approval was obtained (DNR 2020–04246) and all patient data were de-identified prior to analysis to ensure confidentiality and compliance with data protection regulations. The STROBE guidelines were followed [19].

### Setting

The study was conducted using data sourced primarily from the Swedish Trauma Registry (SweTrau) database, a comprehensive repository containing detailed information on trauma patients admitted to hospitals throughout Sweden [20]. The study period spanned over two years between 2018 and 2019.

**Fig. 1:**
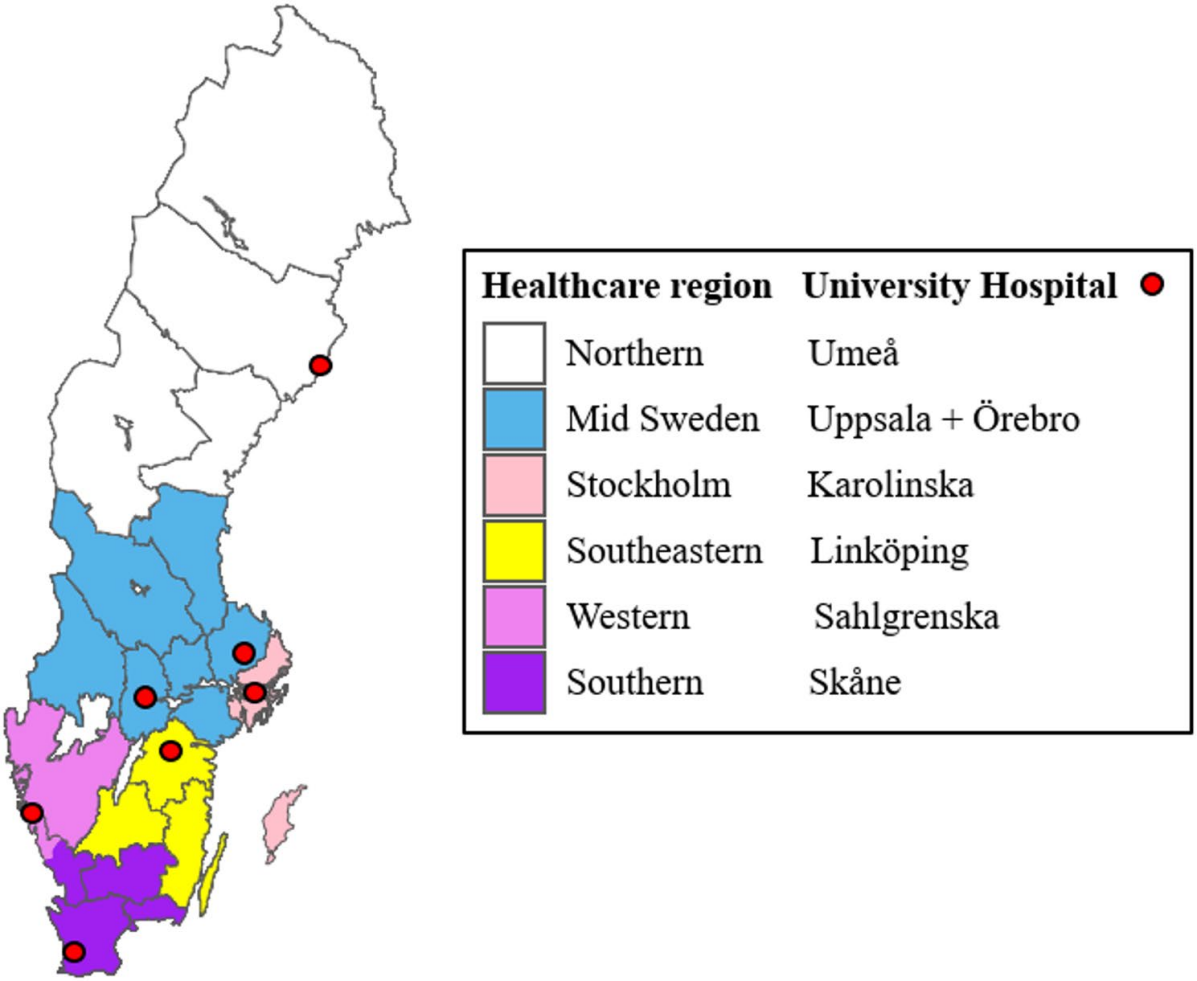
Regions of Sweden grouped by Healthcare regions. The locations of the University Hospitals within each Healthcare region are indicated with red circles

Sweden is a country in northern Europe covering a land area of 410 000 km^2^ with a population during the study period of 10.3 million [21]. The emergency medical service (EMS) system varies across the 21 regions in Sweden with different resource setups. The backbone in all regions are the road ambulances staffed with nurses, while some regions, 9 out of 21, have access to helicopter emergency medical service (HEMS), typically physician staffed [22]. Stockholm and Gothenburg were, at the time of the study, the only two cities with access to physician-staffed rapid response vehicles [23, 24]. The 21 regions of Sweden are independent health care providers and organize the care, including the prehospital resources and setup, themselves. Specialised care such as Neuro or Thoracic surgery and access to the highest level of trauma care is provided through collaboration within 6 Healthcare regions as seen in Fig. 1. Each Healthcare region typically contains one major trauma centre at the University hospital. No national guidelines regarding trauma treatment nor direct referral procedures for trauma patients existed at the time of the study.

### Participants

All patients with complete prehospital time intervals and residential data recorded in SweTrau during the specified study period were included in the study. The inclusion criteria for SweTrau were admissions of trauma patients who at admission to the hospital were received using a trauma call, patients admitted with a new injury severity score (NISS) >15, and patients transferred within 7 days of the trauma with a NISS >15. Exclusion criteria for SweTrau were the cases with chronic subdural hematoma as the sole traumatic injury and if no trauma preceded the trauma call [25, 26].

A predefined subgroup analysis of the more severely injured patients included those with injury severity score (ISS) > 15 was undertaken. SweTrau extracts residential addresses from the Swedish Population Registry using the personal registration number. Patients were grouped by municipalities. Patients admitted to a hospital not located in the same or neighbouring region as the residential address municipality, were excluded. An exception to this rule was applied to patients residing in the three western regions (Värmland, Dalarna and Örebro) of the Healthcare region Mid Sweden who were brought by HEMS to Uppsala University Hospital (located furthest to the east), as these could represent primary admissions to the major trauma centre. Patients with temporal or unknown personal numbers could not be linked to a geographical area. Cases that could not be matched to a geographic area or had missing transport time data were excluded from the analysis. As no transportation time data for the secondary transportations were available in SweTrau, no secondary transports were included in the analysis.

### Variables

Data were extracted from the SweTrau database, which provided information on transportation and response times, patient demographics, injury characteristics, and geographical details. The validity of the SweTrau registry has been described elsewhere [27].

The primary outcome variables were the three components of the total prehospital time; response time, on-scene time and transport time. The response time was defined as the duration between receiving the emergency call and the arrival of the EMS at the accident site. On-scene time was the time from arrival on scene until departure of the EMS. The transport time was defined as the time from scene departure to the patient’s subsequent arrival at the receiving hospital. The total prehospital time was the sum of the three intervals above.

In cases where only a date (and no time) had been recorded in the timestamp of different prehospital stages, the time of the event was automatically set to 00:00; these cases consequently rendered inaccurate calculations for the duration of prehospital time intervals, hence an attempt to systematically validate the recorded timestamps was performed to exclude cases with inaccurate data, as detailed below. The accuracy of prehospital timestamps was assessed for every case, with each timestamp being considered valid if the recorded time was not 00:00. Timestamps with a recorded time of 00:00 were evaluated for accuracy and considered valid if none of the other recorded timestamps had a value of 00:00; cases with only a single recorded timestamp were also considered invalid. Timestamps were then checked for chronological validity and were considered invalid if they violated the chronological order of the prehospital chain of events. Prehospital time intervals were then calculated for each case using only the valid timestamps. Cases with prehospital transport times ≥ 4 h were manually reviewed, excluding those with obvious registration errors (e.g., incorrect date in a single timestamp) from the analysis.

Secondary outcome variables were 30-day mortality, LOS and GOS.

**Fig. 2:**
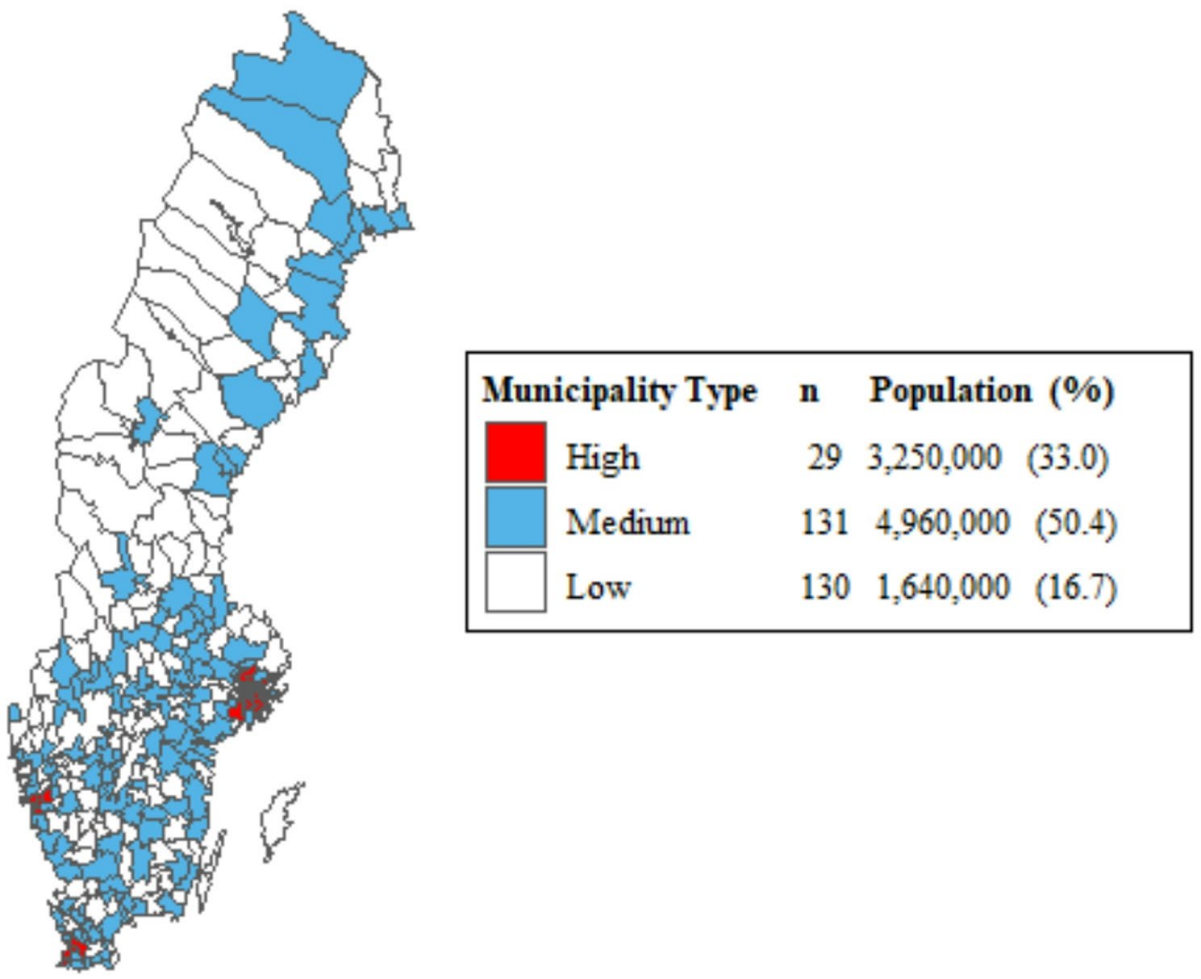
Municipalities of Sweden grouped by population density type, as defined by the Swedish Agency for Growth Policy Analysis. The legend shows the number of municipalities, total population, and the percentage of the national population within each group

Patients were divided into groups according to the population density of their home municipality, classified as high, medium, or low, described by the Swedish Agency for Growth Policy Analysis [28] as metropolitan, mixed and rural municipalities, respectively. See Fig. 2. A detailed description of each group is seen in Table S1 in the appendix.

Other variables of interest were demographics as age, sex, American Society of Anesthesiologists (ASA) score, NISS, ISS, the three components of revised trauma score (RTS) based on Glasgow coma scale (GCS), systolic blood pressure (SBP), respiratory rate (RR), blunt/penetrating trauma, presence of pre-hospital physician, ground/air ambulance, prehospital intubations and intubated if GCS *≤* 8. RTS ranges from 0 to 4 for each category where 4 is the physiological normal value. The lower a RTS, the more deranged the given parameter is. For example RTC SBP of 0 is equivalent to no blood pressure and hence cardiac arrest [29]. A full description of RTS and the corresponding cut-off values are shown in Table S2 in the appendix.

### Bias

The study acknowledges that the injury location may not necessarily correspond to the patient’s residential address, introducing potential bias in geographical representation. In order to address this, the admitting hospital had to be in the same or neighbouring region as the residential address. SweTrau received data from 42 to 43 out of 50 acute trauma receiving hospitals, indicating a reporting ratio of 80–86% [25, 26].

### Statistical methods

Distribution analysis, utilizing histograms and summary statistics, was employed to comprehend the distribution of transport times. The Kruskal-Wallis rank sum test and pairwise Wilcoxon rank-sum test (Mann-Whitney U test) with continuity correction was used to assess the difference in distributions. Bonferroni’s method was used to correct for multiple testing. Differences in proportions were assessed using Pearson’s χ2 test. For non-parametric variables medians were reported with their corresponding interquartile ranges (IQR). For normally distributed variables means and standard deviations were reported. Tests were two-tailed and the significance level of 0.05 was used for all hypothesis tests. If a variable had missing values (MV) these were specified and excluded from the analysis. Multivariate binary logistic regression was used to investigate how age, gender, ISS, ASA, response time, on-scene time, transport time, RTS, physician presence and population density groups were associated with 30-day mortality. The results of the regression analyses were reported as odds ratios (OR) with 95% confidence intervals (CI). In the regression analyses the variables ASA score, RTS and population group belonging, the reference of 1, 4 and low were used, respectively. Statistical analyses were performed, and figures were generated using RStudio ver. 2023.06.1 and PDF-XChange Editor Plus ver. 10.0.1.

## Results

### Participants

**Fig. 3:**
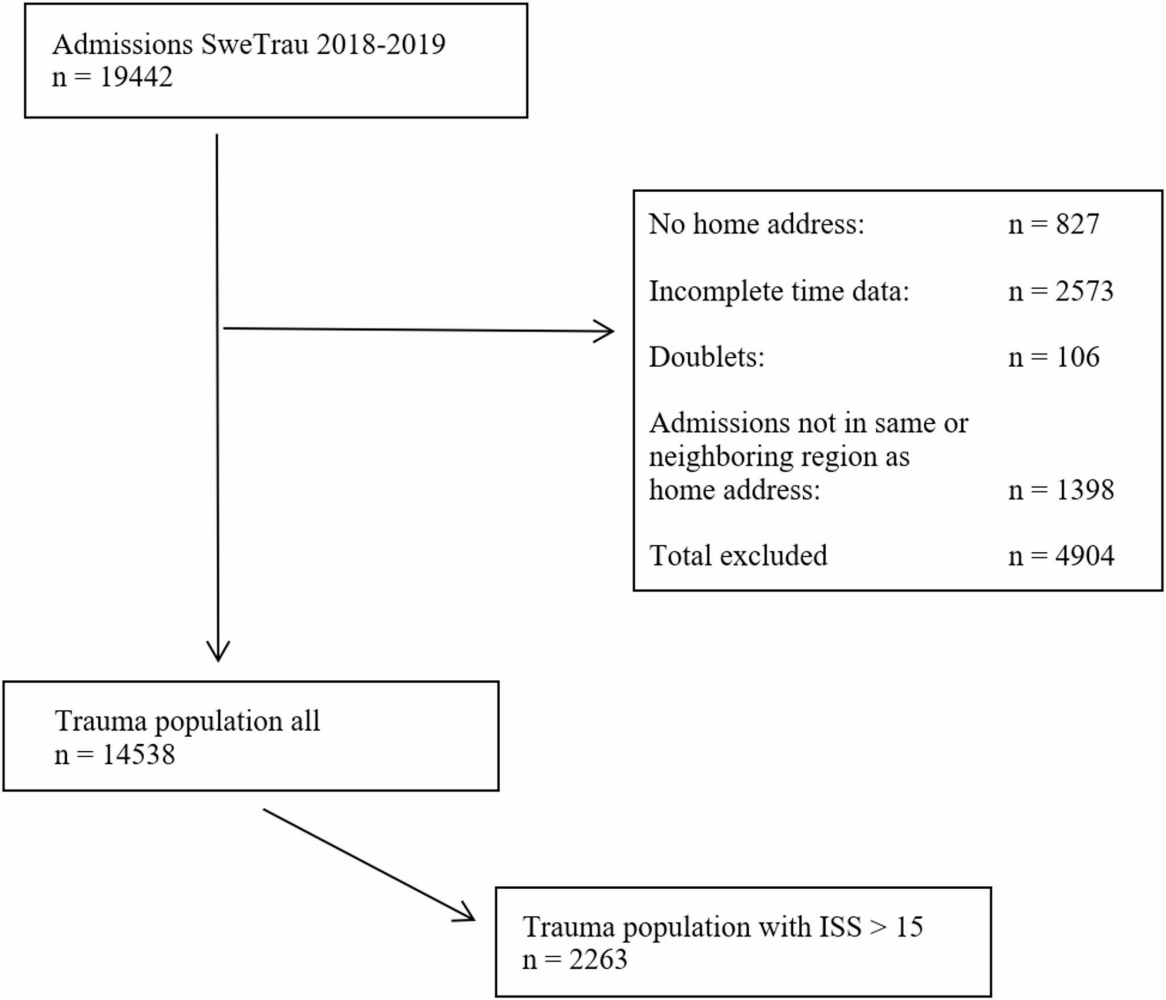
Patient flow chart according to inclusion eligibility. ISS: injury severity score

There were 19,442 registrations in SweTrau during 2018–2019. After exclusions, a cohort of 14,538 admissions with complete prehospital time intervals and classification according to the three population density levels was formed. Seven patients were included due to exemption of the rule of hospital admittance to own or neighbouring region. Sixteen percent had ISS > 15 and hence constituted the corresponding subgroup with 2263 entries, as seen in Fig. 3.

### Demographics and prehospital factors

Demographics, prehospital transport and treatment details are presented in Table 1A and 1B for all patients and the subgroup, respectively. Across the whole injury severity panorama, the patients from the urban group were older, had higher ASA score, higher ISS and were more often intubated. The medians and IQRs for the RTS were uniform across the density groups but showed significant distribution differences. Patients from rural areas were more often transported by air ambulance while the intermediate group to a lesser extent received treatment from a prehospital physician.

**Table 1A:** Demographics, comorbidity, injury description and prehospital information for all trauma patients. IQR: interquartile range, ASA: American society of Anesthesiologists, MV: missing values, ISS: injury severity score, RTS: revised trauma score, GCS: Glasgow coma scale, SBP: systolic blood pressure, RR: respiratory rate

Population density	High	Medium	Low	*p*-value
Patients all, n (%)	4951 (34)	6803 (47)	2784 (19)	
Demographics				
Age in years, median (IQR)	46 (27–67)	41 (22–62)	44 (23–63)	**< 0.001**
Male, n (%)	3169 (64)	4370 (64)	1808 (65)	0.71
Female, n (%)	1782 (36)	2433 (36)	975 (35)	0.71
ASA score, median (IQR) [MV]	1 (1–2) [41]	1 (1–2) [35]	1 (1–2) [16]	**< 0.001**
Physiological and injury parameters				
NISS, median (IQR) [MV]	6 (2–11) [78]	5 (2–10) [10]	5 (2–10) [13]	**< 0.001**
ISS, median (IQR) [MV]	5 (1–11) [78]	4 (1–10) [10]	4 (1–10) [13]	**< 0.001**
ISS 0–15, n (%)	4028 (83)	5768 (85)	2378 (86)	**< 0.001**
ISS > 15, n (%)	845 (17)	1025 (15)	393 (14)	**< 0.001**
RTS GCS, median (IQR) [MV]	4 (4–4) [284]	4 (4–4) [567]	4 (4–4) [366]	**< 0.001**
RTS SBP, median (IQR) [MV]	4 (4–4) [132]	4 (4–4) [242]	4 (4–4) [100]	**< 0.001**
RTS RR, median (IQR) [MV]	4 (4–4) [136]	4 (4–4) [266]	4 (4–4) [139]	**0.03**
Penetrating injury, n (%) [MV]	548 (11) [10]	475 (7) [18]	151 (5) [21]	**< 0.001**
Prehospital factors				
Ground ambulance, n (%)	4688 (95)	6517 (96)	2577 (93)	**< 0.001**
Air ambulance, n (%)	263 (5)	286 (4)	207 (7)	**< 0.001**
Physician present, n (%) [MV]	552 (11) [2]	334 (5) [5]	185 (7) [10]	**< 0.001**
Prehospital intubation, n (%) [MV]	178 (4) [2]	191 (3) [16]	68 (2) [15]	**0.008**
Intubated if GCS *≤* 8, n/total (%) [MV]	137/323 (42) [285]	141/364 (39) [569]	51/127 (40) [368]	0.62

In the subgroup of the more severely injured patients, the high density group had higher ASA score, lower RTS GCS and SBP, higher rate of penetrating trauma and more often received treatment by a prehospital doctor than the other two groups, as seen in Table 1B.

**Table 1B:** Demographics, comorbidity, injury description and prehospital information for patients with ISS > 15. IQR: interquartile range, ASA: American society of Anesthesiologists; MV: missing values, ISS: injury severity score, RTS: revised trauma score, GCS: Glasgow coma scale, SBP: systolic blood pressure, RR: respiratory rate

Population density	High	Medium	Low	*p*-value
Patients ISS > 15, n (%)	845 (37)	1025 (45)	393 (17)	
Demographics and physiological parameters				
Age in years, median (IQR)	55 (32–74)	57 (32–74)	56 (36–71)	0.80
Male, n (%)	593 (70)	729 (71)	295 (75)	0.20
Female, n (%)	252 (30)	296 (29)	98 (25)	0.20
ASA score, median (IQR) [MV]	2 (1–3) [17]	2 (1–3) [17]	2 (1–2) [2]	**0.002**
Physiological and injury parameters				
NISS, median (IQR)	29 (22–41)	27 (22–35)	27 (22–35)	**0.02**
ISS, median (IQR)	22 (17–29)	22 (17–26)	22 (17–29)	0.32
RTS GCS, median (IQR) [MV]	4 (2–4) [62]	4 (3–4) [110]	4 (2.5-4) [58]	**0.03**
RTS SBP, median (IQR) [MV]	4 (4–4) [34]	4 (4–4) [49]	4 (4–4) [15]	**0.002**
RTS RR, median (IQR) [MV]	4 (3–4) [38]	4 (3–4) [64]	4 (4–4) [27]	0.21
Penetrating injury, n (%) [MV]	100 (12) [2]	80 (8) [3]	28 (7) [4]	**0.004**
Prehospital factors				
Ground ambulance, n (%)	754 (89)	918 (90)	338 (86)	0.15
Air ambulance, n (%)	91 (11)	107 (10)	55 (14)	0.15
Physician present, n (%) [MV]	223 (26) [1]	135 (13) [1]	60 (15)	**< 0.001**
Prehospital intubation, n (%) [MV]	135 (16) [1]	138 (14) [5]	44 (11) [5]	0.07
Intubated if GCS *≤* 8, n/total (%) [MV]	107/208 (51) [63]	109/221 (49) [111]	37/82 (45) [60]	0.62

### Prehospital time intervals

The prehospital time intervals for all patients are shown in Table 2A. The median response time was 11 min both in the high and medium density groups which was significantly shorter than the median of 15 min in the low density group. The median on-scene time in the medium group was 20 which was 1 min shorter than group high and low. The transport time shortened with increasing population concentration, which also was the case for the total prehospital time.

**Table 2A:** Prehospital time intervals and secondary outcomes for all trauma patients. IQR: interquartile range, MV: missing values

Population density	High	Medium	Low	*p*-value
Patients all, n (%)	4951 (34)	6803 (47)	2784 (19)	
Time intervals				
Response time in minutes, median (IQR)	11 (7–17)	11 (7–18)	15 (9–23)	**< 0.001**
On-scene time in minutes, median (IQR)	21 (15–29)	20 (14–27)	21 (15–30)	**< 0.001**
Transport time in minutes, median (IQR)	14 (10–20)	19 (10–30)	29 (18–43)	**< 0.001**
Total prehospital time in minutes, median (IQR)	50 (39–63)	54 (39–71)	69 (53–89)	**< 0.001**
Secondary outcomes				
Mortality, n (%) [MV]	310 (6) [7]	372 (6) [57]	123 (5) [47]	**0.005**
Length of stay in days, median (IQR) [MV]	2 (1–5) [4]	2 (1–5) [6]	2 (1–5) [13]	0.82
Glasgow outcome scale, median (IQR) [MV]	4 (4–5) [12]	5 (4–5) [57]	5 (4–5) [32]	**< 0.001**

**Fig. 4:**
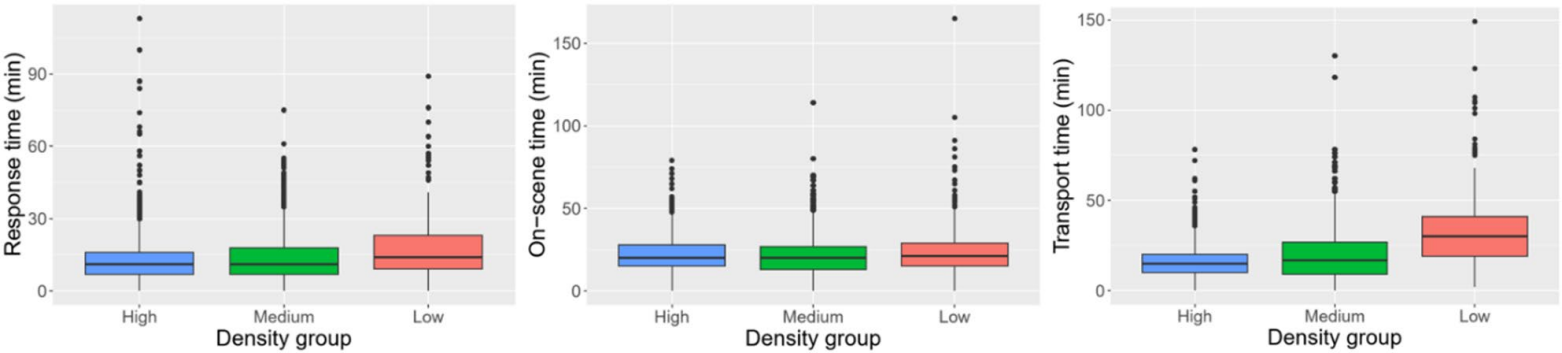
Boxplots showing the distribution of the prehospital time intervals in minutes according to density groups for trauma patients with ISS > 15. ISS: injury severity score

The time intervals in the subgroup of the more severely injured patients, followed the same pattern as the times for the whole trauma population, as seen in Table 2B. The response time was equally 11 min in group high and medium, while 14 min in group low. The median on-scene time was 20 min in both high and medium while 21 min for the group low. Again, the transport time and total prehospital time shortened with increasing population concentration. Figure 4 illustrates the distribution of the three components of the total prehospital time.

**Table 2B:** Prehospital time intervals and secondary outcomes for trauma patients with ISS > 15. IQR: interquartile range, MV: missing values

Population density	High	Medium	Low	*p*-value
Patients ISS > 15, n (%)	845 (37)	1025 (45)	393 (17)	
Time intervals				
Response time in minutes, median (IQR)	11 (7–16)	11 (7–18)	14 (9–23)	**< 0.001**
On-scene time in minutes, median (IQR)	20 (15–28)	20 (13–27)	21 (15–29)	**0.003**
Transport time in minutes, median (IQR)	15 (10–20)	17 (9–27)	30 (19–41)	**< 0.001**
Total prehospital time in minutes, median (IQR)	48 (38–60)	51 (37–68)	70 (52–89)	**< 0.001**
Secondary outcomes				
Mortality, n (%) [MV]	220 (26) [2]	260 (26) [10]	76 (20) [6]	**0.04**
Length of stay in days, median (IQR) [MV]	7 (3–15) [1]	6 (2–16)	6 (2–15)	0.42
Glasgow outcome scale, median (IQR) [MV]	3 (2–4) [4]	3 (2–4) [25]	3 (3–4) [9]	**< 0.001**

### Secondary outcomes

The mortality in the whole population was 6% in high and medium, 5% in the low group, while the GOS was significantly lower in group high versus medium and low. No significant difference in LOS between the three groups was observed. See Table 2A.

In the subgroup of patients with ISS > 15 the mortality was 26% except for the low density group where it was 20%. The difference in LOS distribution was again insignificant in all groups. The median GOS was 3 in all groups but the distribution in the high group was significantly lower than in the other two. See Table 2B.

### Multivariate logistic regression

In the entire trauma population the factors independently associated with increased mortality were age, ISS, ASA score 3 and 4 in relation to 1 and lower RTS, to a lesser extent SBP than GOS and RR. None of the time intervals were associated with mortality. The presence of a prehospital physician was inversely associated with mortality. High population density group was in comparison with low associated with lower mortality, as seen in Table 3A.

**Table 3A:** Multivariate binary logistic regression for 30-day mortality based on all trauma patients (*n* = 12488). CI: confidence interval, ISS: injury severity score, ASA: American society of Anesthesiologists, RTS: revised trauma score, GCS: Glasgow coma scale, SBP: systolic blood pressure, RR: respiratory rate

Variables	Odds ratio	CI 95%	*p*-value
Age	1.07	1.06–1.08	**< 0.001**
Gender	0.86	0.68–1.10	0.24
ISS	1.09	1.08–1.10	**< 0.001**
ASA 2 vs. 1	1.29	0.89–1.88	0.18
ASA 3 vs. 1	2.45	1.69–3.58	**< 0.001**
ASA 4 vs. 1	8.59	4.77–15.34	**< 0.001**
Response time	1.00	0.99–1.01	0.73
On-scene time	1.00	0.99–1.01	0.81
Transport time	1.00	0.99–1.00	0.44
Physician present	0.60	0.39–0.91	**0.02**
RTS GCS 0 vs. 4	21.20	13.51–33.36	**< 0.001**
RTS GCS 1 vs. 4	9.16	4.95–16.84	**< 0.001**
RTS GCS 2 vs. 4	5.53	3.41–8.81	**< 0.001**
RTS GCS 3 vs. 4	3.63	2.52–5.17	**< 0.001**
RTS SBP 0 vs. 4	8.61	2.17–32.47	**0.002**
RTS SBP 1 vs. 4	1.20	0.37–3.62	0.76
RTS SBP 2 vs. 4	1.28	0.54–2.83	0.56
RTS SBP 3 vs. 4	0.90	0.49–1.58	0.73
RTS RR 0 vs. 4	17.59	5.29–65.96	**< 0.001**
RTS RR 1 vs. 4	14.52	5.30–41.19	**< 0.001**
RTS RR 2 vs. 4	1.93	0.68–5.07	0.20
RTS RR 3 vs. 4	2.03	1.46–2.81	**< 0.001**
Density medium vs. low	1.00	0.71–1.44	0.98
Density high vs. low	0.67	0.46–0.99	**0.04**

The results for the corresponding analysis for the subgroup of the more injured patients are seen in Table 3B. Similarly to the results for all trauma patients, none of the time intervals presented any significant association with mortality. Age, ISS, ASA classification 3 and 4 and lower RTS were, also statistically connected to increased mortality. The population density was not itself associated with mortality among the more injured patients.

**Table 3B:** Multivariate binary logistic regression for 30-day mortality based on trauma patients with ISS > 15 (*n* = 1851). CI: confidence interval, ISS: injury severity score, ASA: American society of Anesthesiologists, RTS: revised trauma score, GCS: Glasgow coma scale, SBP: systolic blood pressure, RR: respiratory rate

Variables		Odds ratio	CI 95%	*p*-value
Age		1.05	1.04–1.06	**< 0.001**
Gender		0.96	0.68–1.35	0.81
ISS		1.06	1.04–1.08	**< 0.001**
ASA 2 vs. 1		1.37	0.87–2.17	0.18
ASA 3 vs. 1		2.13	1.32–3.48	**0.002**
ASA 4 vs. 1		10.32	4.12–26.58	**< 0.001**
Response time		1.00	0.98–1.01	0.88
On-scene time		1.00	0.98–1.01	0.62
Transport time		1.00	0.98–1.01	0.47
Physician present		0.57	0.35–0.92	**0.03**
RTS GCS 0 vs. 4		23.80	14.06–41.18	**< 0.001**
RTS GCS 1 vs. 4		7.62	3.80–15.38	**< 0.001**
RTS GCS 2 vs. 4		4.51	2.44–8.26	**< 0.001**
RTS GCS 3 vs. 4		3.37	2.05–5.48	**< 0.001**
RTS SBP 0 vs. 4		5.66	1.20–26.13	**0.03**
RTS SBP 1 vs. 4		0.79	0.23–2.57	0.70
RTS SBP 2 vs. 4		1.13	0.41–2.90	0.81
RTS SBP 3 vs. 4		0.79	0.39–1.53	0.50
RTS RR 0 vs. 4		8.93	2.40–39.50	**0.002**
RTS RR 1 vs. 4		8.62	2.91–27.11	**< 0.001**
RTS RR 2 vs. 4		2.21	0.72–6.53	0.16
RTS RR 3 vs. 4		1.31	0.84–2.02	0.22
Density medium vs. low		1.04	0.64–1.73	0.87
Density high vs. low		0.73	0.43–1.25	0.24

## Discussion

In this retrospective observational cohort study, we found trauma related prehospital time intervals in Sweden to vary across population density groups. The median difference in response time and on-scene time was, however, only 1–4 min, while the transport time difference was more pronounced. Both among all patients, and in a predefined subgroup analysis of the more severely injured patients, where differences in time spent prior to hospital admission reasonably would matter the most, no association between prehospital time intervals and mortality was seen. The mortality was slightly lower in rural areas but after adjustments for covariates, residing in population group high was in relation to group low, in the whole population, associated with better survival.

In contrast, the subgroup with ISS > 15 showed no adjusted association between population density and mortality.

In conclusion, prehospital time intervals were not associated with mortality.

### Prehospital times

The key parameters of interest in this study, response, on-scene and transport times, varied in the three population groups. The response time for all patients, as well as for those with higher ISS, was equally long in high and medium groups, but the increased median in the low population group only differed by 3–4 min. This implies that the overall geographical distribution of EMS stations in Sweden is well balanced, and inhabitants have fairly equal access to EMS attendance. The response time we found was slightly shorter than the median of 15 min for 2021 reported by the National Board of Health [22] for priority 1 tasks. The on-scene time medians only differed 1 min across the groups, also pointing towards equal prehospital trauma care across the population levels. The transport time was the parameter that varied the most across the population groups, which, again reflects the fact that EMS stations in Sweden are spread out, being close to patients, but when there is need for care at centralised hospitals, transport time increases with remoteness.

### Mortality and other secondary outcomes

The patients from the low density group had a lower mortality compared to the high and medium groups. Interestingly, after adjustment for covariates in the multivariate model, for the whole trauma population, there was a tendency towards the opposite as residing in high density areas in comparison to low, was associated with better survival. This was, however, not the case in the more severely injured subgroup, making the results difficult to interpret. As anticipated, age, ISS, a greater comorbidity load and altered physiological parameters were consistently associated with mortality in both models. No link between response time and mortality was seen in either trauma cohort. There was also no association between on-scene duration and mortality which stands in contrast to results from others [7, 16]. However, a systematic review, based on 10 studies, of which most did not show a connection between time spent on scene with increased mortality, even suggests an association between longer on-scene time and improvement in mortality [13].

No correlation between transport time and mortality was seen in our material. An inverse association between transport time and mortality has been reported by others [8, 16], which, could be tempting to interpret as having a longer transport time could be beneficial for survival. However, the anticipated time to reach a hospital could also be considered as a source of possible bias. When a severely ill patient, perhaps in peri-arrest, is located close to a hospital, transportation may be prioritized while the same patient in a remote setting is more likely to be treated on scene due to longer exposure to limited care during transportation. This would in turn introduce selection bias in relation to mortality and transport time. By adjusting for deranged physiology with low level of consciousness, impaired circulation and altered breathing, we aimed to decrease the risk for introducing such bias.

This study adds to previous results showing that trauma patients seem to benefit from the presence of prehospital physicians [24, 30–34]. This finding, which is logical, may not be detectable when adjusting with ISS or NISS only as these are anatomical, lack physiological parameters and can whence be misleading [35]. Nielsbakken et al. [16], who did not include physiological parameters in their multivariate model reported higher odds for mortality when a physician was involved in the prehospital care.

There was no difference in LOS across the density levels. In the urban group the GOS was significantly lower than in the other groups.

### Prehospital factors

Demographic differences across the population groups were present as the high density group was older, had a heavier comorbidity load measured by ASA score, were sicker with lower RTS, displayed a more severe injury panorama, reflected in higher ISS, and more often had penetrating injuries. These tendencies have been described by others in other settings [17]. Physicians prehospitally were most often deployed in urban areas, both in the whole trauma cohort and the ISS >15 group. This finding could be related to the higher intubation frequency in urban areas. Prehospital factors such as more frequent air ambulance usage in rural areas can be explained by aircrafts being effective in remote areas [16].

### Limitations

Although our study provides important insight into prehospital trauma care it has several limitations. The nature of a retrospective cohort study comes with the shortcomings of describing association and not causation. Further, as already stated several types of bias can be present. Also, we compare prehospital systems across 21 independent health care regions in a country characterized by diverse geographical and organisational conditions. In Sweden, there are not yet any overarching national recommendations or guidelines of how or what type of prehospital trauma care should be provided [22]. We used the patient’s home address and not the site of the trauma (as that data was unavailable to us), which introduces some inaccuracy. However, we tried to adjust for this by excluding patients with a residential address not neighbouring the region of the receiving hospital.

## Conclusions

Despite a difference in prehospital time intervals across population density groups, no association between prolonged time spent before hospital admission and increased mortality was seen. Further, residing in either high, medium or low density population areas were not consistently associated with mortality.

## Supplementary Information

Below is the link to the electronic supplementary material.

Supplementary Material 1

## Data Availability

The data can be accessed upon application from the SweTrau organisation [https://swetrau.se/om-swetrau/about-swetrau-in-english].

## Abbreviations

ASA: American Society of Anesthesiologists
CI: Confidence interval
EMS: Emergency medical services
GCS: Glasgow coma scale
GOS: Glasgow outcome scale
HEMS: Helicopter emergency medical service
IQR: Interquartile range
ISS: Injury severity score
LOS: Length of stay
MV: Missing values
NISS: New injury severity score
OR: Odds ratio
RR: Respiratory rate
RTS: Revised trauma score
SBP: Systolic blood pressure
SweTrau: Swedish Trauma Registry
