# Supplementary material for: Prehospital time intervals for trauma patients according to population density levels in Sweden; a national retrospective cohort study
Source: Scand J Trauma Resusc Emerg Med. 2025 Dec 4;33:193. doi: 10.1186/s13049-025-01514-z (PMC12676833; doi:10.1186/s13049-025-01514-z)
Supplement: Supplementary file 1 — Supplementary Material 1 [file 13049_2025_1514_MOESM1_ESM.docx]

## Appendix

##### **Table S1** Definition of the population density groups high, medium and low according to the Swedish Agency for Growth Policy Analysis as metropolitan, mixed and rural municipalities, respectively.

| **Municipality type based on population density** | **Definitions** |
| --- | --- |
| High (metropolitan) | Municipalities with less than 20% of their population in rural areas and a total population of at least 500 000. |
| Medium (mixed) | Other municipalities with less than 50% of their population in rural areas. |
| Low (rural) | Municipalities with at least 50% of their population in rural areas. |

##### **Table S2** Revised trauma score and the physiological parameters defining the score.

| **Revised trauma score component** | **Definitions** |
| --- | --- |
| Glasgow coma scale (GCS) | 4 – GCS 13–15  3 – GCS 9–12  2 – GCS 6–8  1 – GCS 4–5  0 – GCS |
| Systolic blood pressure (SBP) | 4 – SBP > 89 mmHg  3 – SBP 76–89 mmHg  2 – SBP 50–75 mmHg  1 – SBP 1–49 mmHg  0 – SBP 0 mmHg |
| Respiratory rate (RR) | 4 – 10–29 breaths/min  3 – >29 breaths/min  2 – 6–9 breaths/min  1 – 1–5 breaths/min  0 – 0 breaths/min |
